# Transmission and re-infection of Omicron variant XBB.1.5 in hamsters

**DOI:** 10.1016/j.ebiom.2023.104677

**Published:** 2023-06-21

**Authors:** Peter J. Halfmann, Ryuta Uraki, Makoto Kuroda, Kiyoko Iwatsuki-Horimoto, Seiya Yamayoshi, Mutsumi Ito, Yoshihiro Kawaoka

**Affiliations:** a Influenza Research Institute, Department of Pathobiological Sciences, School of Veterinary Medicine, University of Wisconsin–Madison, Madison, WI 53711, USA; b Division of Virology, Institute of Medical Science, University of Tokyo, Tokyo 108-8639, Japan; c The Research Center for Global Viral Diseases, National Center for Global Health and Medicine Research Institute, Tokyo 162-8655, Japan; d Pandemic Preparedness, Infection and Advanced Research Center (UTOPIA), University of Tokyo, Tokyo 162-8655, Japan

**Keywords:** XBB.1.5, Airborne transmission, Animal model, Hamster, Re-infection

## Abstract

**Background:**

Like its predecessors in the XBB family, XBB.1.5 is highly immune evasive from therapeutic monoclonal antibodies and neutralizing antibodies generated by vaccination and/or infection. However, there is a lack of *in vivo* data on XBB.1.5 in animal models such as Syrian hamsters.

**Methods:**

Syrian hamsters (females) were used to examine airborne transmission along with virus replication of XBB.1.5 in naïve animals and human ACE2 hamsters with pre-existing immunity from a previous infection with Omicron BA.1. Assays were performed to determine neutralizing antibody responses, and virus titers were determined by standard plaque assays.

**Findings:**

Unlike earlier Omicron subvariants, such as BA.1 and BA.2, XBB.1.5 transmitted more efficiently in the hamster model. In addition, XBB.1.5 partially escaped BA.1-immunity from a previous infection with XBB.1.5 replicating in the nasal turbinate tissues and to a lesser extend in the lung tissues of previously infected hamsters.

**Interpretation:**

Our *in vivo* data showing better airborne transmissibility of the Omicron subvariant XBB.1.5 than its predecessor, BA.2, in Syrian hamsters will allow researchers to further investigate amino acid substitutions that give XBB.1.5 a fitness advantage over BA.2 in transmission, data that may be important in studies of SARS-CoV-2 transmission in humans.

**Funding:**

This research is supported by grants from the Center for Research on Influenza Pathogenesis and Transmission (CRIPT; 75N93021C00014), funded by the 10.13039/100000060National Institute of Allergy and Infectious Diseases and by a Research Program on Emerging and Reemerging Infectious Diseases (JP21fk0108552 and JP21fk0108615), a Project Promoting Support for Drug Discovery (JP21nf0101632), the Japan Program for Infectious Diseases Research and Infrastructure (JP22wm0125002), and The University of Tokyo Pandemic Preparedness, Infection and Advanced Research Center (UTOPIA) grant (JP223fa627001) from the 10.13039/100009619Japan Agency for Medical Research and Development.

**Research in context**

**Evidence before this study**

Since the emergence of the first Omicron subvariant (BA.1) in November 2021, the Omicron lineage has continued to evolve in the human population, acquiring additional mutations throughout its genome that result in amino acid substitutions in its proteins, including the spike protein. As more substitutions have accumulated in the spike protein, the Omicron subvariants have become more immune evasive to neutralizing antibodies. The XBB.1.5 subvariant is highly immune evasive from therapeutic monoclonal antibodies and neutralizing antibodies generated by vaccination and/or infection. However, there is a lack of information regarding the fitness of XBB.1.5 in an animal model.

**Added value of this study**

In this study, we examined the replication, transmission, and immune escape of XBB.1.5 in Syrian hamsters. We found that XBB.1.5 transmitted more efficiently by droplets than its predecessor, BA.2, which did not transmit at all among hamsters. XBB.1.5 partially escaped BA.1-immunity from a previous infection, with XBB.1.5 replicating in the nasal turbinate tissues and to a lesser extend in the lung tissues of previously infected hamsters.

**Implications of all the available evidence**

Our results suggest a fitness advantage for XBB.1.5 in terms of airborne transmission over the earlier Omicron subvariant BA.2. This information is beneficial to understanding the molecular basis for the airborne transmissibility of SARS-CoV-2.

## Introduction

As of May 3 2023, there have been over 765 million cases of SARS-CoV-2 infection with nearly 7 million deaths around the world.^1^ Because of immune pressures and potentially continued adaptation to a new host (i.e., humans), SARS-CoV-2 continues to acquire mutations throughout its genome that result in amino acid substitutions in its proteins, including the spike protein, the target of approved COVID-19 vaccines. As a result of these amino acid substitutions, a diverse set of SARS-CoV-2 variants have emerged.

In August of 2022, the first XBB subvariants, recombinants between BJ.1 and BM.1.1.1, which both originated from the BA.2 Omicron lineage, were identified.^2^ Further evolution of the XBB lineage resulted in the emergence of the XBB.1.5 variant. Compared to the BA.2 variant, XBB.1.5 has acquired one amino acid deletion (Y145del) and 13 substitutions (V83A, H146Q, Q183E, V213E, G252V, G339H, R346T, L368I, V445P, G446S, N460K, F486P, and F490S) in the spike protein. The F486P substitution in the spike protein of XBB.1.5 provides stronger affinity for human ACE2 compared with the F486S substitution in the spike of XBB.1.^3^ This greater affinity for human ACE2 may contribute to the dominance of XBB.1.5 over other variants circulating in the USA and other parts of the world.

Besides ACE2 affinity, these amino acid substitutions in the spike protein of XBB family members (i.e., XBB, XBB.1, and now XBB.1.5) have also resulted in increased immune evasion from therapeutic countermeasures (i.e., monoclonal antibodies and vaccines).^4^, ^5^, ^6^, ^7^ This loss or reduction of our therapeutic arsenal against COVID-19 highlights the need for animal models in which to evaluate new therapeutics for their ability to reduce the replication and transmission of current and emerging SARS-CoV-2 variants.

However, XBB.1.5 has not been fully characterized in any animal model, including golden Syrian hamsters, which is the only rodent model in which the airborne transmission of SARS-CoV-2 has been effectively evaluated. Hamsters are highly susceptible to the B.1.617.2 (Delta) variant,^8^ but surprisingly, BA.1 variant transmission is greatly reduced in this airborne transmission animal model.^9^ With XBB.1.5 being so prevalent in many countries, knowing whether it has different transmission properties in hamsters compared with earlier Omicron subvariants, such as its predecessor BA.2, would be beneficial to understanding the molecular basis for airborne transmissibility of SARS-CoV-2. In addition, with the continued accumulation of amino acid substitutions during the evolution of the virus, XBB.1.5 may also be able to evade the immunity conferred by previous infection. Therefore, here, we assessed the transmissibility of XBB.1.5 and its ability to establish infection in previously infected hamsters, an established COVID-19 animal model.

## Methods

### Cells and viruses

Vero E6/TMPRSS2 (JCRB 1819) cells were propagated in the presence of 1 mg/ml geneticin (G418; Invivogen) and 5 μg/ml plasmocin prophylactic (Invivogen) in Dulbecco's modified Eagle's medium (DMEM) containing 10% Fetal Calf Serum (FCS). Vero E6-TMPRSS2-T2A-ACE2 cells (provided by Dr. Barney Graham, NIAID Vaccine Research Center) were cultured in DMEM supplemented with 10% FCS, 100 U/mL penicillin–streptomycin, and 10 μg/ml puromycin. Vero E6/TMPRSS2 and Vero E6-TMPRSS2-T2A-ACE2 cells were maintained at 37 °C with 5% CO_2_. The cells were regularly tested for mycoplasma contamination by using PCR and confirmed to be mycoplasma-free.

The B.1.617.2 (hCoV-19/USA/WI-UW-5250/2021), BA.1 (hCoV-19/USA/WI-WSLH-221686/2021), BA.2 (hCoV-2/Japan/UT-NCD1288-2N/2022), and XBB.1.5 (hCoV-19/USA/MD-HP40900-PIDYSWHNUB/2022) viruses were propagated in Vero E6/TMPRSS2 cells.

### Biosafety containment for studies

All experiments with SARS-CoV-2 were performed in enhanced biosafety level 3 (BSL3) containment laboratories at the University of Tokyo and the National Institute of Infectious Diseases, Japan, which are approved for such use by the Ministry of Agriculture, Forestry, and Fisheries, Japan, or in BSL3 agriculture containment laboratories at the University of Wisconsin–Madison, which are approved for such use by the Centers for Disease Control and Prevention and by the US Department of Agriculture.

### Animal studies

Animals were housed for at least 3 days before the start of a study in rooms with controlled temperature and humidity with 12-h light and dark cycles. Animals were monitored at least once daily by trained personnel for any signs of severe infection, a humane endpoint. Food and water were provided *ad libitum* along with enrichment. Virus infections were performed under isoflurane, and all efforts were made to minimize pain. For the animal studies, sample sizes were determined based on prior *in vivo* studies, and no power calculations were performed. Animals were randomly assigned to groups and researchers were not blinded in the selection of the animals. All data were included in the analysis, and no animals or data points were excluded.

Wild-type Syrian hamsters (females; initially 6–8 weeks old; Envigo) were used for virus replication and airborne transmission studies. Animals were infected by intranasal inoculation with 10^5^ plaque-forming units (pfu) of virus in a total volume of 30 μl while under isoflurane anesthesia. For tissue collection, animals were humanely sacrificed by isoflurane overdose. Lung and nasal turbinate tissues were collected for virus titrations; titers were determined by using plaque assays on Vero E6-TMPRSS2-T2A-ACE2 cells.

To evaluate airborne transmission between hamsters, infected donor hamsters were housed in wire cages inside an isolator unit. Twenty-four hours later, naïve hamsters were placed on the other side of the cage. A double-layered wire mesh separated the hamsters by 5 cm to prevent direct contact. The infected, donor hamsters were positioned in the front of the isolator unit, which provided unidirectional airflow. After 48 h of contact time, the hamsters were separated. Tissue samples were collected 3 days after infection for the donor hamsters or 3 days after initial contact for the exposed hamsters.

To evaluated re-infection, K18-hACE2 homozygous transgenic hamsters^10^ were used from an established colony at UW-Madison. Animals (females; 6–8-week-old) were infected by intranasal inoculation with 10^5^ pfu of the Omicron BA.1 variant. Ten months after the primary infection, hamsters (both previously infected and naïve, age-matched controls) were infected with 10^5^ pfu of BA.1 or XBB.1.5. Animals were euthanized by an overdose of isoflurane three days after the secondary infection, and lung and nasal turbinate tissues were collected for virus titration.

### Focus reduction neutralization test (FRNT)

Neutralization of SARS-CoV-2 was characterized in a focus reduction neutralization test (FRNT). Serial dilutions (3-fold) of heat-inactivated hamster sera, starting at a concentration of 1:20, were mixed with approximately 1000 focus-forming units (ffu) of virus/well and incubated for 1 h at 37 °C. The virus-serum mixture was inoculated onto VeroE6/TMPRSS2 cells in 96-well plates for 1 h at 37 °C at which time an equal volume of methylcellulose solution was added to each well. After an 18-h incubation, the cells were fixed with formalin, and then stained with a mouse monoclonal antibody against the nucleoprotein of SARS-CoV (clone 1 C7C7, Sigma–Aldrich; RRID:AB_2893327) followed by incubation with a secondary horseradish peroxidase labeled IgG antibody (Sera Care Life Sciences). The number of stained foci was quantified by using the ImmunoSpotS6 analyzer.

### Statistics

**Fig. 1:**
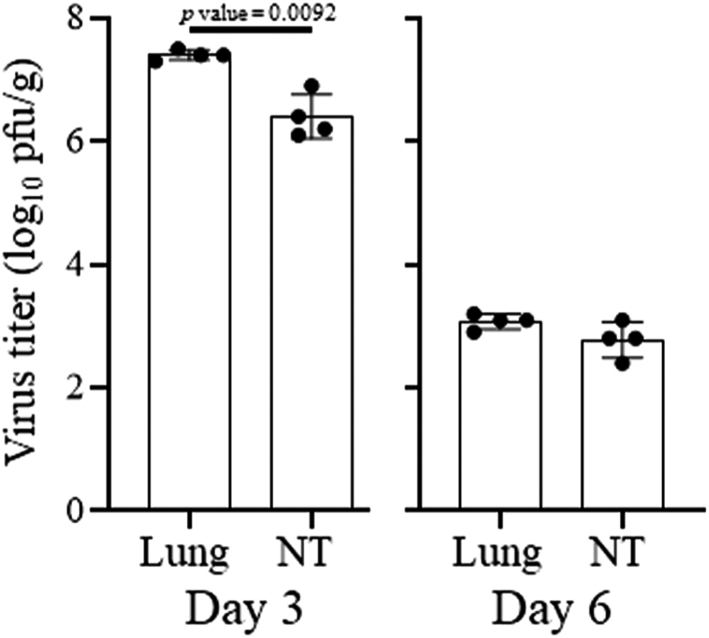
**XBB.1.5 replication in wild-type hamsters.** Virus titers on Days 3 and 6 after infection in the lung and nasal turbinate tissues [mean with SD, n = 4 (Welch's t-test)].

**Fig. 2:**
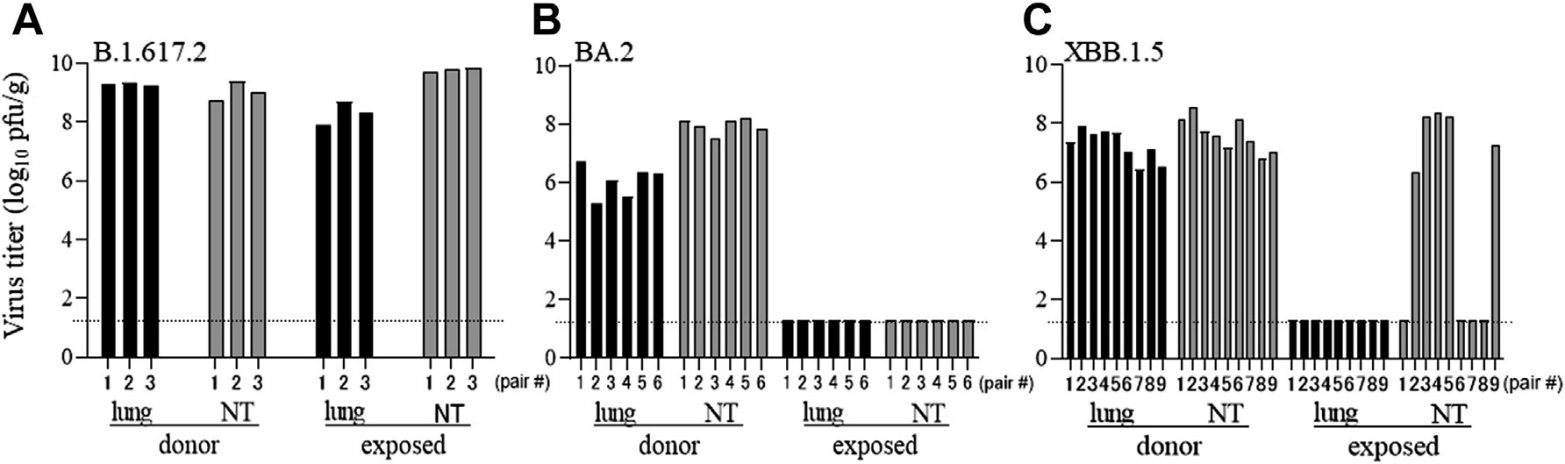
**Airborne transmission of B.1.617.2, BA.2, and XBB.1.5 in Syrian hamsters.** Virus titers in the lung and nasal turbinate (NT) tissues of infected hamsters and exposed hamsters. Transmission was evaluated for (**A**) B.1.617.2 (3 pairs of hamsters), (**B**) BA.2 (6 pairs of hamsters), and (**C**) XBB.1.5 (9 pairs of hamsters). Virus titers are indicated by single bars for each hamster. BA.2 and XBB.1.5 transmission was compared by using Fisher's exact test, *p* value = 0.044). Virus titers are expressed as plaque-forming units per gram (pfu/g). The dotted line indicates the limit of detection.

**Fig. 3:**
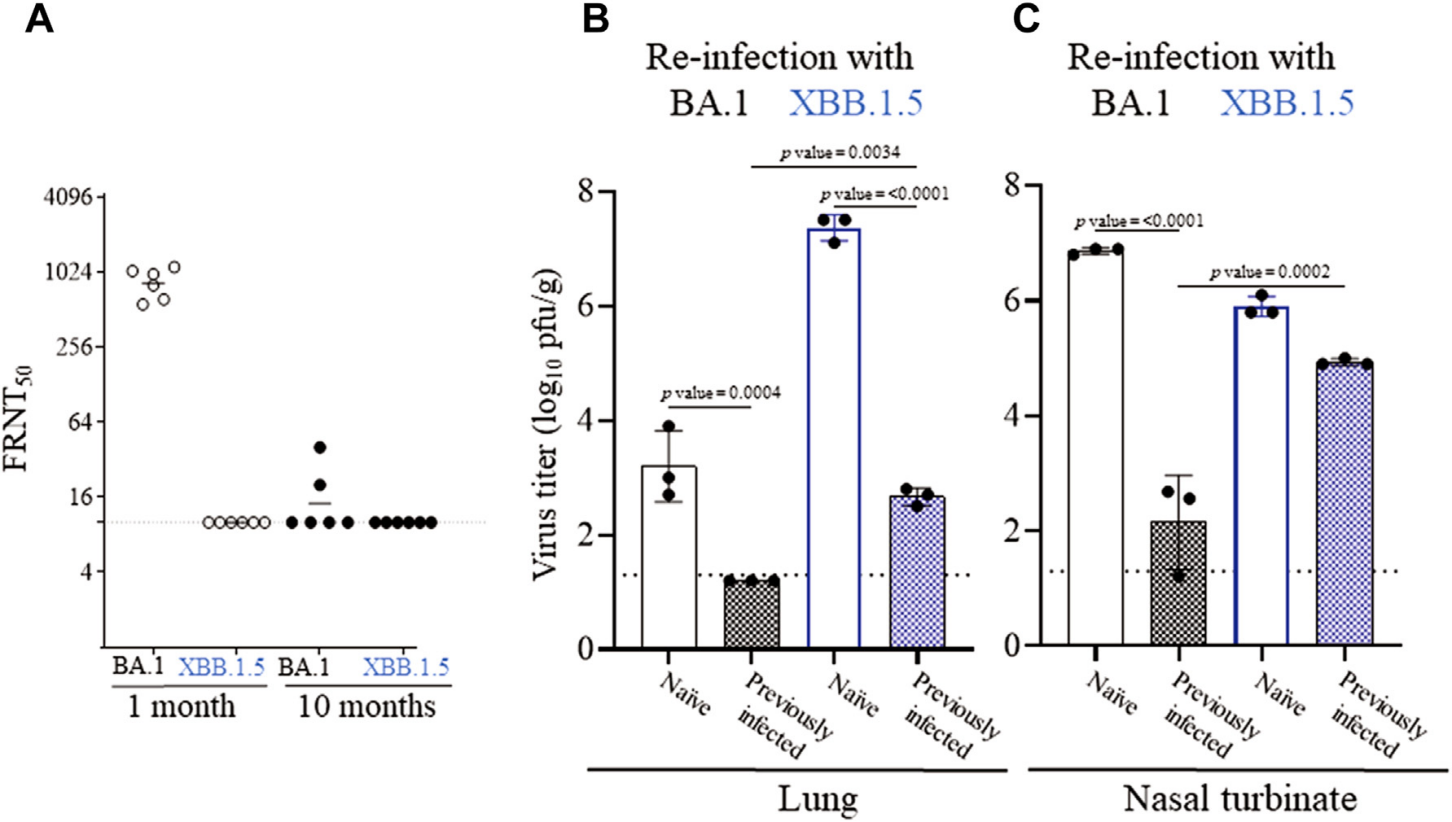
**BA.1 and XBB.1.5 replication in hACE2 hamsters. (A)** Neutralizing antibody titers against BA.1 and XBB.1.5 using hamster serum taken at 1 or 10 months after an infection with BA.1. Virus titers on Day 3 after (re)-infection in the (**B**) lung tissues or (**C**) nasal turbinate tissues of naïve hamsters (n = 3 for each variant) and hamsters previously infected with BA.1 (n = 3 for each variant) after (re)infection with BA.1 (black) or XBB.1.5 (blue). Virus titers are expressed as plaque-forming units per gram (pfu/g). The dotted line indicates the limit of detection. [mean ± SD, n = 3 for each group (multiple comparison, one-way ANOVA)].

Virus titers in the lung and nasal turbinate tissues on each day are presented as the mean with SD (n = 4); the significance was determined by using Welch's t-test with a *p* value indicating whether the difference in virus replication in the two tissues was significant (*p* value < 0.05; Fig. 1). Virus titers from each hamster in the transmission study are shown as a single bar in the group with the number of pairs listed (Fig. 2). To compare transmission between BA.5 and XBB.1.5, a Fisher's exact test calculation was performed. To determine significance between naïve hamsters and hamsters previously infected with BA.1, a one-way analysis of variance (ANOVA) with Tukey's multiple comparisons test was performed (Fig. 3B and C). GraphPad Prism 9 software was used to perform the analysis.

### Ethics

The animal study protocols were approved prior to the start of the studies by the Animal Experiment Committee of the Institute of Medical Science, the University of Tokyo (approval number PA19-75) and the Institutional Animal Care and Use Committee at the University of Wisconsin, Madison (protocol number V006426).

### Role of funders

The funders of the study had no role in the study design, data collection, analysis, interpretation, or the writing of this report.

## Results

### Virus replication and airborne transmission of XBB.1.5 in hamsters

To assess the levels of infection in the respiratory tract of wild-type Syrian hamsters, animals (n = 4 for each day, females; 6–8 weeks old) were intranasally inoculated with 10^5^ plaque-forming units (pfu; 30-μl total volume) of XBB.1.5. At 3 and 6 days after infection, lung and nasal turbinate tissues were collected for virus titration on Vero E6-TMPRSS2-T2A-ACE2 cells using a standard plaque assay.

On Day 3 after infection, XBB.1.5 replicated to higher virus titers in the lung tissues compared to the nasal turbinate tissues (*p* value = 0.0092), reaching mean titers of 7.4 and 6.4 log_10_ pfu/g, respectively (Fig. 1). On Day 6 after infection, virus titers were similar in both tissues (no significant difference) and were reduced by 3–4 log units compared with the earlier timepoint (Fig. 1).

With the global spread of XBB.1.5, we also examined the transmission of XBB.1.5 compared to other variants in hamsters (females; 6–8 weeks old) by intranasally inoculating them with 1 × 10^5^ pfu (30-μl total volume) of B.1.617.2 (hCoV-19/USA/WI-UW-5250/2021), BA.2 (hCoV-2/Japan/UT-NCD1288-2N/2022), or XBB.1.5 (hCoV-19/USA/MD-HP40900-PIDYSWHNUB/2022). Infected donor and naïve exposed hamsters were paired together for 48 h to allow virus transmission.

Similar to our previous data, transmission of our positive control variant, B.1.617.2, was observed in all three pairs of hamsters (100% transmission) (Fig. 2A). No infectious BA.2 virus was detected in any of the six exposed hamsters (0% transmission) even though the six infected, donor hamsters had average virus titers of 10^6^ and 10^8^ pfu/g in their lung and nasal turbinate tissues, respectively (Fig. 2B). For XBB.1.5, infectious virus was detected in the nasal turbinate tissues in five of nine exposed hamsters (56% transmission), but infectious virus was not detectable in the lung tissues of the exposed hamsters (Fig. 2C), demonstrating that XBB.1.5 is more transmissible than BA.2 in hamsters (*p* value = 0.044, Fisher's exact test).

### Re-infection of hamsters with XBB.1.5

With the immune evasion properties of XBB.1.5, we examined the ability of XBB.1.5 to re-infect hamsters previously infected with BA.1. In this study, transgenic human ACE2 hamsters were first infected with 1 × 10^5^ pfu of BA.1 (hCoV-19/USA/WI-WSLH-221686/2021). One month after the primary infection, hamsters had high circulating neutralizing antibody titers against BA.1 in their serum compared with XBB.1.5. Neutralizing antibody titers were nearly undetectable against both variants at 10 months after infection (Fig. 3A).

Therefore, 10 months after the first infection, the previously infected (n = 3 for each variant) or age-matched naïve hamsters (n = 3 for each variant) were (re-)infected with BA.1 or XBB.1.5. In the lung tissues, replication of BA.1 and XBB.1.5 was better in the naïve hamsters than the previously infected hamsters (*p* values = 0.004 and < 0.0001, respectively (Fig. 3B). However, infectious XBB.1.5 virus was detected in the lung tissues of all three previously infected hamsters, albeit to a lesser extent, compared with the replication of the variant in naïve hamsters (Fig. 3B). The ability of XBB.1.5 to overcome immunity from a previous infection in the lung tissue was statistically significant compared to the inability of BA.1 to re-establish an infection (*p* value = 0.0034).

In the nasal turbinate tissues, BA.1 replicated significantly better in the naïve hamsters than in the previously infected hamsters (*p* value = <0.0001), whereas there was no statistically significant difference between the replication of XBB.1.5 in the two different groups of hamsters (naïve and previously infected; Fig. 3C). Again, the ability of XBB.1.5 to overcome immunity from a previous infection was better than the ability of BA.1 to re-establish an infection (*p* value = 0.0002).

## Discussion

The first Omicron variant, BA.1, emerged in November 2021, with at least 33 substitutions, deletions, and insertions within the spike protein.^11^ While these changes resulted in increased immune evasion of this variant compared with early SARS-CoV-2 isolates,^12^^,^^13^ there was a cost in fitness in rodent models of SARS-CoV-2 infection. Early Omicron variants such as BA.1, BA.2, and BA.5 were attenuated in mice and hamsters.^14^, ^15^, ^16^, ^17^ In addition, there was no detectable airborne transmission of BA.1 in the hamster model.^9^

However, the Omicron lineage has continued to evolve in the human population acquiring additional amino acid substitutions throughout its genome including the spike protein. With the addition of these genomic changes in the more recent XBB Omicron subvariants, there is evidence of an increase in viral fitness, as seen with the XBB subvariant.^18^ The more recent XBB family member XBB.1.5 has acquired 14 amino acid changes in the spike protein compared to its earlier predecessor, BA.2. These changes in the viral genome may contribute to the overall fitness of XBB.1.5 as it replicates better in the respiratory tissues (lung and nasal turbinate) of hamster compared to past Omicron subvariants such as BA.1 and BA.2.^14^^,^^17^

In addition, these substitutions in the XBB family, including XBB.1.5, have resulted in further loss of effective therapeutic antibodies and reduced vaccine efficacy, with these family members being the most resistant SARS-CoV-2 variants to date.^7^ This resistance includes the loss of all clinical monoclonal antibodies^4^, ^5^, ^6^ as well as significant reductions in serum neutralizing antibodies including those induced by the bivalent booster vaccine.^7^

Immunity induced by a prior SARS-CoV-2 infection has protected hamsters from re-challenge in the hamster model, although our data shows that this immunity is lessened with an XBB.1.5 challenge. In a previous study, a prior Omicron BA.1 infection (5-weeks prior) prevented virus replication in the lung and nasal turbinate tissues after re-inoculation with BA.1 or pre-Omicron variants such as B.1.1.7, B.1.351, or B.1.617.2.^19^ In addition, there is little effect on lung pathology after re-infection with different SARS-CoV-2 variants including BA.1 in hamsters.^20^

In the present study, we also examined re-infection after a primary infection with BA.1. In humans with underlying medical conditions such as obesity, diabetes, or hypertension, re-infection with SARS-CoV-2 has been observed 38–87 days after the primary infection.^21^ A larger systematic meta-analysis suggested that re-infection with SARS-CoV-2 ranges from 90 to 650 days after a primary infection, with an average of 343 days or about 11 months.^22^ Therefore, in this study, we re-challenged hamsters 10 months after the primary infection, when circulating neutralizing antibodies in the serum were low to undetectable. In contrast to previous reports, here we detected infectious, replicating XBB.1.5 in the lung tissues of hamsters that had preexisting immunity due to a prior infection, demonstrating that immunity from prior infection is less effective against XBB.1.5. These data corroborate similar reports of reductions in serum neutralization of XBB family members with human sera from individuals receiving the monovalent mRNA vaccine series including those who also received the bivalent booster.^6^^,^^7^

More striking was the change in the transmission fitness of XBB.1.5 in naïve hamsters. In the hamster model, our data show that XBB.1.5 transmits more efficiently than BA.1^9^ and BA.2 in this study, two Omicron subvariants that lacked an airborne transmission phenotype in hamsters. It is difficult to compare transmission properties of XBB.1.5, or any other SARS-CoV-2 variant, between hamsters and humans. In humans, the data suggest a high dispersion rate of SARS-CoV-2 with 20% of infected individuals potentially responsible for 80% of virus transmission.^23^^,^^24^ Attempts to experimentally recreate such human transmission scenarios in animal models such as hamsters are problematic given biosafety containment factors and the cost of such studies. Therefore, continued in-depth epidemiological studies to characterize chains of viral spread in the human population are essential to help shape public health and control measures.

Overall, many factors contribute to the differences between these two susceptible hosts, including the different immune states in the human population along with potential anatomic and physiological differences. It is unclear why Omicron subvariants do not transmit efficiently in hamsters by droplets yet spread quickly in the human population. It is reminiscent of influenza virus and the ferret model in that not all human influenza virus isolates transmit efficiency in ferrets relative to the efficient transmission seen in humans.^25^

Ultimately, hamsters are a model and one tool that researchers can use to help answer questions about infectious diseases such as COVID-19 that may translate to human health. Here, we demonstrated that in this animal model, XBB.1.5 shows increased viral fitness, potentially due to newly acquired mutations in its spike gene or elsewhere in the genome. In this context, the hamster model may contribute to a better understanding of the molecular basis for SARS-CoV-2 airborne transmissibility, virus replication, and immune escape.

## Contributors

P.J.H., R.U., M.K., K.I.-H., S.Y. and M.I. planned the studies, carried out the hamster experiments, and analyzed the data. P.J.H. and Y.K. obtained funding, conceived the study, wrote the draft manuscript with all other authors providing editorial comments. P.J.H. and Y.K. have accessed and verified the underlying data reported in the manuscript. All authors read and approved the final version of the manuscript.

## Data sharing statement

Data supporting the findings of this study are present in the paper and/or Supplementary Materials. Additional data related to this paper may be requested from the authors.

## Declaration of interests

Y.K. has received unrelated funding support from Daiichi Sankyo Pharmaceutical, Toyama Chemical, Tauns Laboratories, Inc., Shionogi & Co. LTD, Otsuka Pharmaceutical, KM Biologics, Kyoritsu Seiyaku, Shinya Corporation, and Fuji Rebio. The remaining authors declare that they have no competing interests.
